# Rearing the Cotton Bollworm, *Helicoverpa armigera*, on a Tapioca-Based Artificial Diet

**DOI:** 10.1673/031.007.3501

**Published:** 2007-05-23

**Authors:** Bilal Haider Abbasi, Khalique Ahmed, Feeroza Khalique, Najma Ayub, Hai Jun Liu, Syed Asad Raza Kazmi, Muhammad Nauman Aftab

**Affiliations:** ^1^ National Key Laboratory of Biochemical Engineering, Institute of Process Engineering, Graduate University of Chinese Academy of Sciences, Beijing-100080, PR China.; ^2^ Department of Biological Sciences, Quaid-i-Azam University, Islamabad-45320, Pakistan; ^3^ Pulses Program, Crop Sciences Institute, NARC, Park Road, Islamabad, Pakistan; ^4^ College of Biological Sciences and Biotechnology, Beijing Forestry University, Beijing-100083, China; ^5^ State Key Laboratory of Computer Science, Institute of Software, Graduate University of Chinese Academy of Sciences, Beijing-100080, PR China; ^6^ Bioinformatics Laboratory and National Laboratory of Biomacromolecules, Institute of Biophysics, Graduate University of Chinese Academy of Sciences, Beijing-100101, PR China

**Keywords:** diet cost efficiency, life history, Manihot esculenta

## Abstract

The impact of a tapioca-based artificial diet on the developmental rate, life history parameters, and fertility was examined over five consecutive generations for the cotton bollworm, *Helicoverpa armigera* Hubner (Lepidoptera: Noctuidae), a highly polyphagous pest of many agricultural crops. The study showed that when fed the tapioca-based artificial diet during larval stage, larval and pupal developmental period, percent pupating, pupal weight, emergence rate of male and female, longevity, fecundity and hatching were non-significantly different than that of the control agar-based artificial diet. Moreover, the cost to rear on tapioca-based diet approached 2.13 times less than the cost of rearing on the agar-based artificial diet. These results demonstrate the effectiveness and potential cost savings of the tapioca-based artificial diet for rearing *H. armigera*.

## Introduction

The cotton bollworm, *Helicoverpa armigera* Hubner (Lepidoptera: Noctuidae) is a major threat to intensive agriculture (Sigsgaard et al. 2002). Its wide dissemination and pest status has been attributed to its polyphagy, and its ability to undergo both facultative diapause and seasonal migration (Fitt 1989). The species is migratory on all continents, and is a key pest on all of them (Feng et al. 2005). Host plants used by *H. armigera's* have been recorded for India (60 cultivated and 67 wild plants) (Karim 2000), Africa (Pearson 1958), Australia (Zalucki et al. 1986), and New Zealand (Thanee 1987).

It is important to be able to economically rear important insects to study their life history, behavior, feeding habits, and their susceptibility and resistance to chemical and biological pesticides. Rearing insects on artificial diets is an expensive process, especially for developing countries where insufficient funds are available for research. As a result it is inevitable that economic threats imposed by insects to agriculture are poorly studied (Ahmed 1983). So far various artificial diets have been developed and proposed for the maintenance, and continuous rearing of economically important insects (Cohen, 2001; Castane and Zapata 2005; Ahmed et al. 1998). Although there is some success in efforts to rear successive generations of economically important insects entirely on an artificial diet, in many cases there is loss of both fitness and reproductive potential which cause longer development times and lower fecundity (Coudron et al. 2005). As a result, the cost-saving ratio is diminished. Life and fecundity tables have been found to be important methods for analyzing and understanding the impact of an external factor, such as an artificial diet, upon the growth, survival, reproduction, and rate of increase of an insect population (Bellows et al. 1992). These tools have been used to improve rearing techniques (Birch 1948) and compare different food sources in diet (Hansen et al. 1999).

**Table 1.**
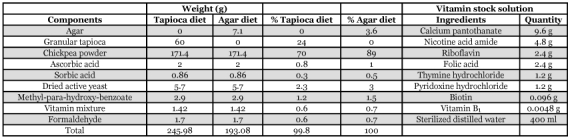
Ingredients of agar and tapioca-based diets and vitamin solution.

Agar is a vital ingredient of insect rearing diet (Ahmed et al. 1998) and is acquired from marine algae such as *Gracilaria* and *Geladiella* species (Nene 1996). Worldwide, agar is the most expensive and sole ingredient of biological media. It is characterized as a gelling agent and provides some minerals and probably provides a stimulation of gut motility, which can be very important in terms of absorption of nutrients and effective digestion (Cohen, 2003). There are other functions of diet components such as modifiers of bioavailability, stability, palatability, emulsification, and other aspects of hydrocolloid function such as viscosity, sheer strength and tensile strength (Cohen, 2003). Tapioca, prepared from the cassava plant, *Manihot esculenta* Crantz (Euphorbiales: Euphorbiacea) has been successfully used instead of agar in plant tissue culture media (Nene 1996; Gebre and Sathyanarayana 2001). Tapioca can also be used as a gelling agent in media. In this report we show that tapioca can be used in place of agar to rear *H. armigera* for up to five successive generations. All materials used in this study were fabricated locally with the purpose of determining cost effectiveness when compared to imported materials from other countries. The impact of the tapioca-based artificial diet was studied on larval development, pupal development, pupal weight, incomplete pupation, sex emergence percentage, fecundity and longevity, which were compared with results of simultaneously reared consecutive generations on the agar-based diet formula.

## Materials and Methods

A colony of *H. armigera* was initiated from 2 pairs of adults collected from the wild environment and reared on the agar-based diet used as the control in current study (Ahmed et al. 1998). Experiments were conducted on the second generation of that wild collected pair. The first experimental generation reported in results was the third generation of that wild pair. The experimental conditions were kept at 70+5% RH, 25±1°C with a photoperiod of L:D 16:8.

**Table 2.**
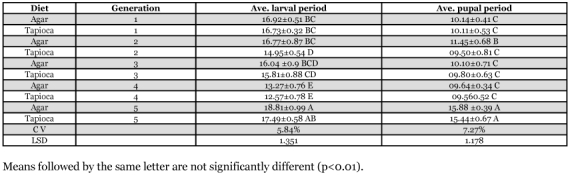
Average larval and pupal periods for *H. armigera*

### Ingredients of the diet

The composition of the diet is shown in Table 1. The wet and dry ingredients of the diet were weighed and kept separately. The agar or tapioca was suspended in 3.5 liter of water and boiled. For the tapioca-based diet a heat-proof mixer from Braun was used while boiling to ensure complete mixing and grinding of the tapioca. The chickpea powder was then added to the boiled mixture and mixed, during this process the temperature of the mixture became nearly 60°C. The remaining dry and wet ingredients were then added to the mixture with thorough mixing.

### Ingredients of the vitamin stock solution

Table 1 also shows the composition of the vitamin mixture. All dry ingredients were added to a flask and sterilized distilled water was gradually added with steady stirring until the entire quantity of powder had been dissolved. Water was then added until the 400 ml volume was obtained.

### Egg incubation

Eggs were collected from the lab colony on layers of cotton wool oviposition pads and enclosed in polyethylene bags. Eggs were allowed to develop at room temperature. After the larvae started hatching they were transferred to glass vials containing the diet.

### Glass capsule vial technique for individual larval development

To study the larval stage a glass vial (2.5cm in diameter and 5.5cm in height) was used. Diet (7 ml) was placed into sterilized vials and a newly hatched first instar larva was added using a camel hairbrush. In order to provide an air exchange a sterilized cotton wool plug was used which also prevented drying of the diet before the developing larva pupated. Four replicates of 25 vials each for agar and tapioca-based diets were run simultaneously.

### Adult emergence

Adult emergence was studied using plastic Petri plates (1.5cm high with 9cm diameter). After larvae pupated, the pupae were placed on a circular piece of blotting paper in the Petri dish with 1 pupa in each plate. After emergence, the adults were placed in individual vials for egg laying. The details of cages have been described previously (Ahmed et al. 1998). Briefly, medium sized lamp glasses 10.2 cm high having 7.9 cm lower end and 6.6 cm upper end diameter were used as oviposition cages for single pairs of adults, and plastic jars having 10 cm lower end and 12cm upper end diameter were used as oviposition cages for two to three pairs of adults.

### Statistical analysis

The data were subjected to a one-way analysis of variance (ANOVA). Tukey-Kramer Test was used for calculation of significant differences. By using SPSS (for Windows, standard version 7.5.1 by SPSS Inc. Chicago), P<0.01 value was regarded as significant. The data compared were within generations of similar treatments.

## Results and Discussion

The results show that tapioca is a suitable alter native for *H. armigera* continuous rearing up to five generations. Table 1 shows that by replacing 7.1 g of agar with 60 g of tapioca, the total concentration of each remaining ingredient changed by about 20%. In the tapioca based diet, for every gram of diet that larva consume, it acquire nearly 20% less protein than it was ingesting with the agar diet, (chickpea powder and yeast are the only significant sources of protein in both formulations). The same reduction also occurs for the lipid content, the vitamins, etc., but the carbohydrate composition increases. The water content goes from 84% of the agar diet to about 80% of the tapioca diet, which is another potentially important change that may affect insect growth (Cohen, 2003). The nutritional importance of tapioca contributes little beyond carbohydrates. Thus, although tapioca does have a little more nutritional value than agar, it does not make up the nutritional gaps.

**Table 3.**
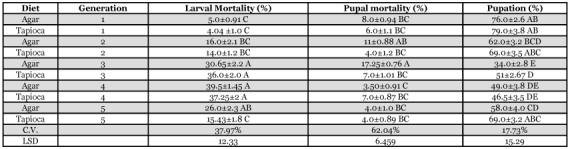
Percentage larval mortality, pupal mortality, and pupation of *H. armigera*.

In order to compare effects of diet on the biology of *H. armigera* two groups were raised on agar-based and tapioca-based artificial diets. It was found that the average larval period of the 4^th^ and 5^th^ generations on either agar or tapioca based diet showed significant differences with other generations (Table 2). By the 5^th^ generation non-significant differences were observed between tapioca and agar-based diets, indicating that they were nutritionally equivalent. These results agree with Ahmed (1983). The 5^th^ generation also showed a significantly longer average pupal development period on both diets.

The results in Table 3 indicate that larval mortality varied considerably during the generations, with little significant differences between diets. The variation in morality from 1^st^ to 5^th^ generation may be linked to the factors vis-à-vis larval entanglement in the cotton plug, or injury from infestation or fungal contamination. Sutter and Muller (1971) reared army cutworm with 3.2% larval mortality, and Ahmed (1983) reported 2.7% mortality in 1^st^ generation *H. armigera*, which were reared on bean powder diet.

**Table 4.**
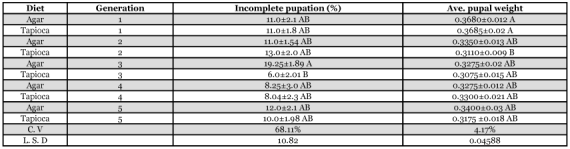
Incomplete pupation and pupal weight of *H. armigera*.

By the 4 and 5^th^ generations pupal mortality was not significantly different between diets (Table 3). The highest mortality of 17.25% was recorded for the 3 generation fed the agar-based diet. The high pupal mortality was attributed to incomplete chitinization of the first three abdominal segments on the ventral surface (cause unknown), and fungal contamination. The 3rd generation of *H. armigera* reared on a bean powder diet exhibited high pupal mortality of 62.88%, and this value was linked with incomplete chitinization of segments of pupa (Ahmed et al. 1998; Howell, 1972).

Percent pupation varied considerably over the 5 generations with no significant differences between diets, except for the 3^rd^ generation. Maximum pupation of 79% was observed for the 1^st^ generation on tapioca-based diet while its minimum value was 34% for the 3^rd^ generation on the agar-based diet (Table 3). Significant differences were observed in percent pupating for the 3rd generation on the agar-based diet when compared with other generations on the same diet.

**Table 5.**
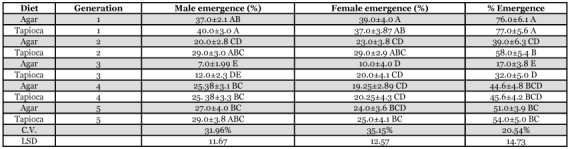
Percent male emergence, female emergence and total emergence of *H. armigera*.

When *H. zea* was reared on bean and wheat-soy blend diet, the pupation achieved was 86.2% and 92% respectively (Burton and Perkins, 1972). Whereas, maximum pupation was 83.7% for the 1^st^ lab generation and 61.64% for the 1st field generation for *H. armigera* (Ahmed et al. 1998). The sorghum stem borer, *Chilo zonellus*, was successfully reared on Kabuli gram diet (Dang et al. 1970), with 75% pupation, which is similar to 79% pupation for the 1^st^ generation reared on the tapioca-based diet (Table 3).

The incidence of incomplete pupation varied non-significantly over 5 generations, except for the 3^rd^ generation (Table 4). Entanglement of larva in cotton plugs was major reason causing incomplete pupation. The average pupal weights were also not significantly different between generations.

The percent adult emergence was significantly higher for the 1^st^ generation on both diets but varied considerably during the 5 generations (Table 5). No abnormalities were observed for any generation. The minimum adult emergence was 17.0% for the 3^rd^ generation on the agar diet which was significantly different from those fed the tapioca-based diet. Male and female emergence varied during the generations, but by the 4^th^ and 5^th^ generations they were not significantly different (Table 5).

**Table 6.**
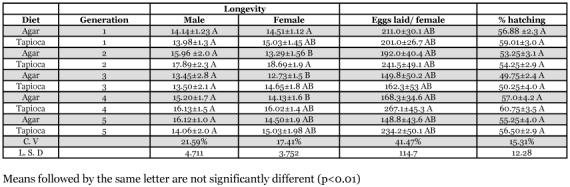
Oviposition/ female, longevity of male and female and percentage hatching of *H. armigera*.

Longevity of males and females was not significantly affected by diet with the exception of the second generation (Table 6). The number of eggs laid was not significantly affected by diet. However, the number of eggs laid by females fed the tapioca diet was always higher than those fed agar-based diet. The number of eggs laid generally increased for those fed the tapioca diet, and fell for those fed the agar diet. A correlation between the length of life of the female and the number of eggs laid is suggested by the data in Table 6. These non-significant differences may be important over long term rearing. The percent of eggs hatching was not significantly affected by diet. The vigor and viability of the insects was normal up to eight generations (data not shown).

The maximum number of eggs laid by a female (326.6) was recorded by Ahmed et al. 1998 for 3^rd^ generation of *H. armigera* on an agar-based diet. Burton (1970) reported rearing of *H. zea* on a corn-soybean meal-based diet and recorded an average oviposition of 406 eggs for mated females. Burton and Perkins (1972) found 1901 eggs from a female *H. zea* reared on a wheat-soy blend diet. Egg production was affected by temperature and was adversely affected by higher temperature, which was probably due to inhibition of mating and oviposition (Ahmed et al. 1998; Urbaneja et al., 2002; Kersting et al., 1999).

A quantitative examination of eggs laid throughout the oviposition period was done by Hou and Sheng (1999) who suggested that the increase in egg deposition in multiple-mated females may be related to hormonal effects on egg production. It has been observed that repeatedly mated females had short lives (LaMunyon 1997). Male mating frequency in *H. armigera* had a dominating effect on fecundity of the paired females, and fecundity also affected the life span of adult females (Hou and Sheng 1999).

It is evident that agar is the most expensive ingredient of insect rearing diet, and consequently its substitution would have the great est effect. The average cost estimated for production of one pupa was Rs. 0.59 (USD 1= PKR 60) on the tapioca-based diet, while the cost on the agar-based diet Rs. 1.24. This reduced the price for tapioca-based diet by 2.13 fold. A 10% sucrose solution was used to feed adults that cost Rs. 3.0 for one generation. Shorey and Hale (1965) reported that the approximate cost to produce a *H. zea/H. virescens* pupa was equivalent to Rs. 0.70 using an agar-based diet, and Ahmed et al. 1998 produced pupa on a modified agar-based diet for Rs. 1.4. The diet developed herein is more efficient and feasible for short and long term rearing of *Helicoverpa armigera*.

The tapioca-based diet developed for rearing *H. armigera*, maintained this insect for up to eight generations with no loss of vigor or viability. This diet would, therefore, have potential to be used as an artificial diet for rearing several other economically important Lepidoptera. This is a good beginning for a new diet for this polyphagous insect. Further improvements for the formulation and production are feasible, and this would likely augment the cost-effective use of the diet for mass rearing of *H. armigera*.
